# Rehabilitation for Functioning and Quality of Life in Patients with Malignant Pleural Mesothelioma: A Scoping Review

**DOI:** 10.3390/curroncol31080322

**Published:** 2024-07-30

**Authors:** Lorenzo Lippi, Alessandro de Sire, Vittorio Aprile, Dario Calafiore, Arianna Folli, Fjorelo Refati, Andrea Balduit, Alessandro Mangogna, Mariia Ivanova, Konstantinos Venetis, Nicola Fusco, Marco Invernizzi

**Affiliations:** 1 Department of Scientific Research, Off-Campus Semmelweis University of Budapest, Campus LUdeS Lugano (CH), 1085 Budapest, Hungary; lorenzolippi.mt@gmail.com; 2 Department of Medical and Surgical Sciences, University of Catanzaro “Magna Graecia”, 88100 Catanzaro, Italy; 3 Research Center on Musculoskeletal Health, MusculoSkeletalHealth@UMG, University of Catanzaro Magna Graecia, 88100 Catanzaro, Italy; 4 Department of Surgical, Medical, Molecular Pathology and Critical Area, University of Pisa, 56126 Pisa, Italy; 5 Department of Neurosciences, ASST Carlo Poma, 46100 Mantova, Italy; 6 Department of Health Sciences, University of Eastern Piedmont “A. Avogadro”, 28100 Novara, Italy; 7 Institute for Maternal and Child Health, Istituto di Ricovero e Cura a Carattere Scientifico (IRCCS), Burlo Garofolo, 34100 Trieste, Italy; 8 Institute of Pathological Anatomy, Department of Medicine, University of Udine, 33100 Udine, Italy; 9 Division of Pathology, European Institute of Oncology, IRCCS, 20141 Milan, Italy; 10 Department of Oncology and Hemato-Oncology, University of Milan, 20122 Milan, Italy; 11 Translational Medicine, Dipartimento Attività Integrate Ricerca e Innovazione (DAIRI), Azienda Ospedaliera SS. Antonio e Biagio e Cesare Arrigo, 15121 Alessandria, Italy

**Keywords:** malignant pleural mesothelioma, physical function, muscle, complementary treatment, physical exercise, rehabilitation

## Abstract

Malignant pleural mesothelioma (MPM) represents a significant clinical challenge due to limited therapeutic options and poor prognosis. Beyond mere survivorship, setting up an effective framework to improve functioning and quality of life is an urgent need in the comprehensive management of MPM patients. Therefore, this study aims to review the current understanding of MPM sequelae and the effectiveness of rehabilitative interventions in the holistic approach to MPM. A narrative review was conducted to summarize MPM sequelae and their impact on functioning, disability, and quality of life, focusing on rehabilitation interventions in MPM management and highlighting gaps in knowledge and areas for further investigation. Our findings showed that MPM patients experience debilitating symptoms, including fatigue, dyspnea, pain, and reduced exercise tolerance, decreasing quality of life. Supportive and rehabilitative interventions, including pulmonary rehabilitation, physical exercise improvement, psychological support, pain management, and nutritional supplementation, seem promising approaches in relieving symptoms and improving quality of life but require further research. These programs emphasize the pivotal synergy among patient-tailored plans, multidisciplinary team involvement, and disease-specific focus. Despite advancements in therapeutic management, MPM remains a challenging disease with limited effective interventions that should be adapted to disease progressions. Rehabilitative strategies are essential to mitigate symptoms and improve the quality of life in MPM patients. Further research is needed to establish evidence-based guidelines for rehabilitative interventions tailored to the unique needs of MPM patients.

## 1. Introduction

Malignant pleural mesothelioma (MPM) is a rare and aggressive cancer occurring in 5–6/100,000 patients per year and is associated with a poor prognosis [1]. MPM is responsible for more than 40,000 deaths per year worldwide [2,3], with an overall survival estimated between 13.3 and 20.2 months [4]. The incidence of MPM is still rising due to the extensive use of asbestos during the past century, which is considered the main risk factor for MPM and is related to over 90% of cases [3,5].

Despite advances in therapeutic management, the prognosis for MPM remains poor. While chemotherapy, specifically multitargeted antifolate together with a platinum compound, is currently considered the first-line treatment [6,7,8,9,10,11,12,13], and immunotherapy is emerging as a promising option [14], the focus of this review is on supportive and rehabilitative interventions that aim to improve quality of life (QoL) for MPM patients [15,16,17,18,19,20].

Patients with MPM often suffer from severe symptoms such as dyspnea, frequently related to malignant pleural effusion [21], and intense pain due to direct invasion of the pleura or chest wall [22]. Additionally, those undergoing MPM surgery commonly experience exercise intolerance and pulmonary function impairments, which negatively impact health-related quality of life (HRQoL) [23]. Despite these significant challenges, the disabling sequelae of MPM and their detrimental effect on HRQoL are often underestimated and poorly addressed, likely due to the rarity of the disease and its poor prognosis [23].

Research on supportive care for MPM patients is limited, with most studies characterized by small sample sizes, hindering the ability to draw firm conclusions about the efficacy of these interventions. The gap in knowledge about MPM-specific rehabilitative interventions, combined with the lack of dedicated clinical pathways, has resulted in rehabilitation approaches that rely on evidence derived from other lung and thoracic cancers.

Given the substantial clinical, emotional, and social burdens of MPM, prompt and effective management of its disabling sequelae is crucial to improving HRQoL for both patients and caregivers. Physical activity and rehabilitation interventions have been widely proposed as effective non-pharmacological therapies in managing cancer-related functional and disabling sequelae [24,25]. However, there is still a lack of consensus about specific rehabilitative exercise protocols tailored for MPM patients. Most of the current literature assesses rehabilitation protocols in lung cancer generally [26,27], and few studies provide data on interventions specifically for MPM patients.

Therefore, the aim of this narrative review is to summarize the main MPM sequelae and their impact on functioning, disability, and HRQoL. Moreover, this review aims to summarize current evidence on rehabilitation interventions for MPM patients to potentially guide future research and improve clinical management of MPM-related symptoms.

## 2. Materials and Methods

### 2.1. Literature Strategy

A scoping review design and methodology was used due to the exploratory nature of the research question. The systematic review followed the recommendations of the Preferred Reporting Items for Systematic Reviews and Meta-Analyses (PRISMA). The protocol has not been registered. We conducted literature searches of the PubMed/Medline, Scopus, Web of Science (WoS), and Physiotherapy Evidence Database (PEDro) electronic databases using the following keywords: “Mesothelioma”, “Rehabilitation”, and “Quality of Life”. The search was conducted by humans and English-language peer-reviewed publications. 

### 2.2. Study Identification

**Figure 1:**
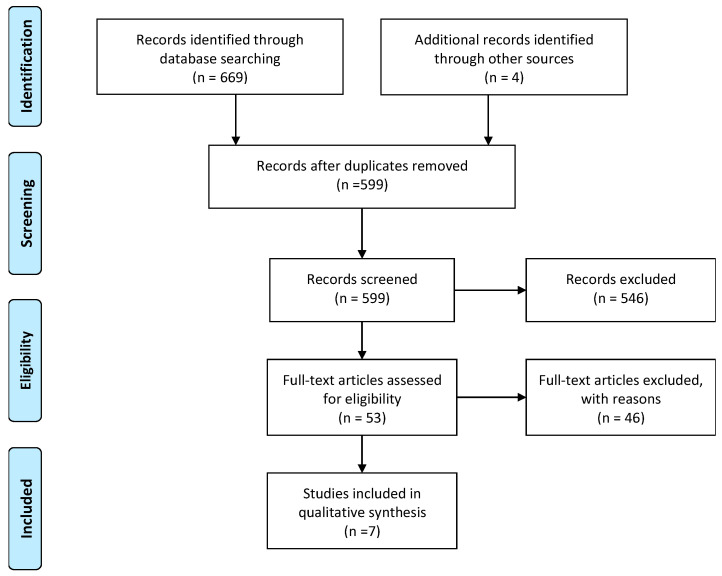
PRISMA flow diagram, to illustrate the process of identification, screening, eligibility, and inclusion of sources.

Two independent reviewers (L.L. and D.C.) performed a literature search between 1 September 2022 and 29 February 2024 and screened the studies for eligibility, reviewing all titles and abstracts identified from the search strategy. In agreement with the predefined eligibility criteria, full-text studies for all potentially eligible records were obtained; accordingly, with the previously decided eligibility criteria, the reviewers independently revised the bibliography. If a consensus was not reached by collegial discussion, a third reviewer (A.d.S.) was asked. More details are shown in Figure 1. 

## 3. Mesothelioma

### 3.1. Predictive Factors and Prognosis

Prognostic markers play a key role in MPM management, providing prospective information on the clinical evolution of patients, thus guiding therapeutic decisions with personalized and tailored treatments. In particular, several prognostic factors have been identified in order to better stratify the prognosis of MPM patients based on both clinical and pathological characteristics [28]. In this scenario, clinical stage and histology are currently considered the most reliable prognostic factors, with sarcomatoid and biphasic histologic subtypes related to worse prognosis, while the pure epithelioid ones related to better outcomes [29]. Other clinical characteristics associated with poor prognosis were summarized in Table 1 [30].

Different prognostic scoring systems, such as EORTC, CALGB, and Brims Prognostic Index, have been proposed for assessing the prognosis of patients with malignant pleural mesothelioma (MPM), but they lack molecular biomarkers [30,31,32]. However, recent studies have identified potential molecular biomarkers, particularly those related to inflammation, such as fibroblast growth factor binders, thrombospondin-1, vascular endothelial growth factor, basal lamina reduplication, cyclooxygenase-2 (COX-2) overexpression, and programmed death-ligand 1 (PD-L1) [33,34,35]. MET protein and EZH2 expression are also implicated in prognosis, with MET protein being suggested as a predictive marker for platinum-pemetrexed chemotherapy. Low thymidylate synthase (TS) protein levels correlate with disease control after carboplatin/pemetrexed therapy [36,37]. Serum soluble mesothelin-related peptides (SMRP) are useful for therapy monitoring and MPM diagnosis [38,39,40]. BAP1 germline mutations have been identified as a positive prognostic factor [41].

### 3.2. Therapeutic Interventions

Chemotherapy, particularly cisplatin with pemetrexed therapy, has been shown to improve survival in MPM. Carboplatin is an alternative for patients’ intolerance to cisplatin [13,42,43]. Nivolumab plus Ipilimumab combination therapy has shown significant improvements in overall survival (OS) compared to standard chemotherapy. Bevacizumab supplementation to cisplatin and pemetrexed, and the use of tumor-treating fields (TTFields), which interfere with mitosis, are additional options in the first-line setting. Second-line therapy remains challenging, but the combination of ramucirumab and gemcitabine or nivolumab is supported in the literature [44,45,46].

Radiotherapy in malignant pleural mesothelioma (MPM) is used for focal palliation and in multimodality regimens after cytoreductive surgery, but its impact on survival is uncertain. It is also used palliatively to reduce chest wall masses or alleviate pain [47,48,49,50,51,52]. Pleural effusion, a common complication, is often managed with talc pleurodesis, but its effectiveness is limited, leading to the proposal of alternatives like indwelling pleural catheters (IPCs) [53,54,55]. Radical surgery like extra-pleural pneumonectomy (EPP) does not offer benefit within trimodal therapy anymore, with pleurectomy decortication (P/D) being the most common surgical procedure. Extended P/D is performed when diaphragm or pericardium resection is necessary. The clinical benefit and cost-effectiveness of (extended) P/D are still uncertain [16,56,57].

The therapeutic options for patients suffering from MPM are reported in Table 2.

### 3.3. Main Disabling Sequelae

Patients with advanced thoracic cancers, often combined with several pulmonary and/or cardio-vascular comorbidities (e.g., chronic obstructive pulmonary disease (COPD)), frequently exhibit physical symptoms responsible for altered quality of life (QoL), reduced physical activity, and a decline of their exercise capacities during chemotherapy [58,59,60]. Patients with MPM are characterized by early and severe symptom burden, mainly including fatigue, cough, and dyspnea. Associated pleural effusions, circumferential tumor growth around the lung, and chest wall expansion are often responsible for such invalidating symptoms. Additional symptoms may include chest pain, lethargy, and weight loss, all of which can negatively impact HRQoL due to impaired physical, mental, emotional, and social functioning. Therefore, early supportive care interventions are essential to counteract all these adverse effects and to maintain a good HRQoL as long as possible [61]. Even though few data are available on HRQoL, a potentially useful tool is represented by remote symptom monitoring, proven to be effective in the management of people with cancer being monitored at home. A specific promising system for MPM has been recently developed (i.e., Advanced Symptom Management System (ASyMS)) [62]. In 2018, QoL data were extracted from 17 articles (14 datasets) encompassing 659 patients (102 EPP, 432 P/D). Despite the limited and low quality of the data, this review pointed out that HRQoL was still compromised 6 months after surgery. HRQoL outcomes should be factored into the choice of surgical procedure for MPM patients, and the possible effects on lung function and HRQoL should be discussed with patients when presenting surgical treatment options [63]. Similar findings about lung cancer function and HRQoL in patients with MPM undergoing P/D have been published more recently [64]. Moreover, a study examining HRQoL in MPM patients receiving chemotherapy has demonstrated that 92% of patients have three or more physical symptoms at presentation [65]. Fatigue (94%), dyspnea (89%), appetite loss (87%), and pain (85%) were accounted as the most common symptoms, with fatigue, dyspnea, and pain associated with a worse overall HRQoL. RESPECT-meso was a multicenter randomized study evaluating the role of early specialist palliative care (SPC) on HRQoL in MPM patients. This led to a post hoc exploratory analysis of the symptom burden and unmet needs of 174 participants using the General Health Status (GHS) measure (from the EORTC QLQ-C30 QoL questionnaire) and 87 participants using validated assessment questionnaires in those randomized to SPC. At least three symptoms were reported in 69.8% of participants, including fatigue (81%), dyspnea (73.3%), pain (61.2%), and weight loss (59.3%), thus highlighting a high symptom burden in MPM associated with worse HRQoL and poor survival, despite good baseline PS [66]. Pulmonary rehabilitation is a core component of managing individuals with chronic respiratory disease and is associated with significant improvement of symptoms, physical activity level, and HRQoL [67]. However, few studies have been performed on advanced-stage cancers so far [68]. This would be very useful in MPM patients who experience an early lung hypoexpansion due to talc pleurodesis, extensive P/D, or simply to the burden of the disease itself, with symptoms having a detrimental effect on HRQoL.

A summary of the main disabling sequelae can be found in Table 3. 

## 4. Quality of Life and Rehabilitative Interventions

### 4.1. Quality of Life, Functioning, and Disability in MPM Patients

Besides the therapeutic interventions to improve the OS, scientific literature has demonstrated an accumulating interest in unveiling the complex management of cancer-related symptoms. To date, it has been reported that patients suffering from thoracic cancer might be less physically active than healthy individuals, with detrimental consequences in terms of muscle strength, nutritional status, and HRQoL assessed by EORTC QLQ-C30-LC13 [59]. More in detail, nutritional status, body composition, and HRQoL measured by SF-36 have been evaluated in the recent study by Jeffery et al. [69], where a homogeneous cohort of 61 patients suffering from MPM was prospectively evaluated. The authors reported that 38% of patients were malnourished and 54% were pre-sarcopenic. Moreover, patients with malnutrition had a significantly lower HRQoL than well-nourished participants [mean 69.0 (16.3) vs. 84.4 (13.3); *p* < 0.001]. These MPM-specific data are crucial in view of the recent evidence underlining the potential role of malnutrition in the OS rate of cancer patients. In particular, a recent meta-analysis performed by Zhang et al., involving 4692 cancer patients, reported that malnutrition was significantly associated with an increased risk of mortality (RR: 1.73; 95% CI: 1.23–2.41). Concurrently, it has been hypothesized that malnutrition and weight loss may be strictly related to the inflammatory process underpinning MPM pathogenesis, considering the crucial role of chronic local inflammation and asbestos-related cytokine dysregulation [70]. Therefore, it is not surprising that fatigue has been accounted for among the most commonly reported symptoms in MPM patients [71]. Kao et al. reported a significant correlation between fatigue and several inflammatory markers (C-reactive protein, NLR, and interleukin-6) in MPM patients. Moreover, the inflammatory systemic status was significantly related not only to HRQoL but also to OS in these patients [71]. In light of these considerations, a comprehensive rehabilitative approach, involving both nutritional supplements and physical exercise, has been proposed as an effective therapy to mitigate the adverse effects associated with several cancer conditions [72,73,74,75]. However, the current literature suggested the existence of several barriers to physical exercise in MPM patients, underlining the worsening of pulmonary function after lung resection, characterized by lower forced expiratory volume in 1 s (FEV1) and forced expiratory vital capacity (FVC) [76,77,78]. In contrast, while the surgical approach may have a role in pulmonary functional impairment, a study by Marulli et al. [79] reported that induction chemotherapy might be related to a significant improvement of FEV1 (0.13 ± 0.30; *p* = 0.01) and VO2 peak (1.76 ± 2.91 mL kg^−1^ min^−1^; *p* = 0.005). Furthermore, in concert with functional impairment, thoracic surgery approaches might have detrimental consequences on QoL. In this scenario, a recent systematic review assessed the effects of MPM surgery on QoL, reporting intriguing results [63]. The authors analyzed 17 articles involving over 600 patients, which showed a consistent deterioration of QoL at 6 months after surgery. Furthermore, *P*/D was related to better QoL, physical function, social function, and global health compared to other surgical procedures. Conversely, a study by Ambrogi et al. [80] reported a deterioration of FEV1 (*p* = 0.06) and FVC (*p* = 0.09) after thoracic surgery (EPP) combined with adjuvant therapies (chemotherapy and radiotherapy), whereas the 6-min walk test, pain, dyspnea, SF-36 physical and mental components, and St. George’s Respiratory Questionnaire symptom significantly improved (*p* < 0.05) at the 3-month follow-up evaluation. However, a progressive worsening has been demonstrated in the long-term follow-up in both functional and SF-36 parameters. The authors suggested that interventions aiming at improving functional parameters might have a role not only in managing MPM functional consequences but also in MPM patient survival. In line with these findings, a recent systematic review and meta-analysis by Nakano et al. [81], including 26 observational studies, underlined the strict relation between physical function and mortality in cancer patients. In particular, mortality risk was significantly associated with handgrip strength test (HR = 1.15, *p* = 0.005), gait speed (HR = 1.58, *p* = 0.0004), short physical performance battery (SPPB) (HR = 2.37, *p* < 0.00001), 6-mine walking test (6MWT; HR = 1.55, *p* = 0.001), and timed up and go (TUG) test (HR = 2.66, *p* < 0.00001). Taken together, these findings highlighted the need for specific rehabilitative interventions in the therapeutic pathway of MPM to mitigate the impaired functional outcomes and QoL due to both cancer itself and active treatments. In this complex scenario, cancer rehabilitation has been currently recognized as a crucial player in improving functional and performance outcomes, with benefits in terms of systemic inflammation, osteoporosis, fatigue, and QoL in other cancer diseases [25,82,83,84,85]. However, to date, good-quality studies are mandatory to provide specific evidence in patients suffering from MPM.

### 4.2. Respiratory Interventions

Respiratory rehabilitative interventions are a crucial part of treating MPM symptoms [86]. Thus far, few studies [87,88] assessed the effects of pulmonary rehabilitation in a homogeneous cohort of MPM patients. Therefore, rehabilitation programs currently used in clinical practice are mainly derived from the evidence collected in lung cancer [89]. More specifically, it has been proposed that pulmonary rehabilitation for patients with MPM should be based on the following three key concepts: (i) patient-tailored rehabilitation plan; (ii) rehabilitation intervention involving a multidisciplinary team; and (iii) rehabilitation focused on the specific characteristics of the disease [89]. A comprehensive assessment, including an evaluation of functional and performance status, dyspnea, pain, fatigue, and HRQoL, should be performed with specific measurement tools to provide clinically relevant data in order to guide rehabilitation prescription [89]. Despite the pivotal role of pulmonary rehabilitation, few studies addressed this issue involving both high-stage lung cancer and MPM patients [90,91,92,93,94,95,96]. To the best of our knowledge, the first report focusing on pulmonary rehabilitation in MPM patients was performed in 1999 by Breading et al. [90]. In this multicenter randomized controlled trial (RCT), the authors assessed the efficacy of a comprehensive intervention, including breathing control, activity pacing, relaxation techniques, and psychosocial support, in patients with lung cancer or MPM, and they reported positive effects in terms of breathlessness, PS, and physical and emotional states. Similarly, Hately et al. [91] assessed the effects of three sessions of 90 min over four to six weeks in patients with lung cancer and MPM. According to the previous evidence, breathlessness was significantly improved after the intervention (*p* < 0.05). Moreover, a significant enhancement was also recorded in terms of functional capacity, activity levels, and distress levels. Taken together, these findings highlighted the positive role of pulmonary rehabilitation in an integrated therapeutic pathway to manage disabling sequelae in lung and MPM cancer patients. However, the optimal and precise respiratory intervention programs (i.e., type, frequency, intensity, and duration) remain controversial. In a randomized controlled non-blinded parallel group feasibility study, Barton et al. [92] assessed the effects of three breathlessness management training sessions compared to a single session only in patients suffering from lung cancer or MPM. Despite the authors not reaching significant results, likely due to the small sample size, potential positive effects were reported in the three-session group in terms of the numerical rating scale of breathlessness severity and distress. Similarly, another multicenter RCT by Johnson et al. [93] explored the effects of a comprehensive respiratory intervention characterized by breathing training, anxiety management, relaxation, pacing, and prioritization. The intervention group received three sessions per week, while the control group had just one session. The authors did not find any significant between-group differences; they concluded that one session per week might be cost-effective in minimizing the symptom burden in patients with lung cancer or MPM. To date, respiratory training devices are frequently used in inspiratory muscle training, with some evidence supporting their potential role in a comprehensive rehabilitative intervention in patients with COPD [97]. However, only one study assessed the effects of specific inspiratory muscle training in a heterogeneous cohort composed of both lung cancer patients and MPM [94]. The patients underwent five sessions of 30 min of inspiratory muscle training per week for a total of 12 weeks. Intriguingly, the authors reported a significant improvement in the inspiratory training group, considering breathlessness (*p* = 0.03), fatigue (*p* = 0.005), emotional function (*p* = 0.011), and depression (*p* = 0.028). In conclusion, despite the limited number of studies investigating the effects of rehabilitative respiratory interventions in MPM patients [90,91,92,93,94], it should be noted that currently available data concern heterogeneous samples composed of both lung cancer and MPM patients. In addition, the effects of rehabilitative respiratory interventions were mainly evaluated in combination with several interventions. Thus, further studies are warranted to guide rehabilitative respiratory intervention prescription and to optimize symptom management in MPM patients. 

### 4.3. Physical Exercise Interventions

Over the past few years, several papers highlighted the central role of physical exercise in the complex management of the most common disabling symptoms related to cancer and its active treatments. It has been widely recognized that physical exercise may improve muscle mass, muscle strength, and physical function [95,98,99], which are frequently impaired in patients suffering from MPM [81]. In light of these considerations, physical exercise is supposed to positively mitigate the most common symptoms complained by patients with MPM. However, there is a large gap of knowledge about this issue in the current literature. In greater detail, most of the studies supporting physical exercise interventions in MPM patients were performed in samples including patients with advanced lung cancer [68,87,100,101,102]. Indeed, specific data on patients suffering from MPM are still lacking. In 2013, Jacobsen et al. [100] assessed the effects of a comprehensive intervention composed of both self-directed stress management training and home-based exercise on quality of life in cancer patients receiving chemotherapy. They found a significant improvement in anxiety and depression symptoms, supporting the role of a multimodal and multidisciplinary approach to improve cancer patients HRQoL. In 2018, Olivier et al. [68] evaluated the outcomes of a home-based exercise intervention combined with therapeutic education and psychosocial management in patients with lung cancer and MPM. After the intervention, the results showed a significant improvement in terms of 6MWT in the MPM subgroup (*p* <0.01), whereas additional benefits were shown in the whole sample in terms of physical performance evaluated by the 10 Chair Stands (10CS) test (*p* = 0.04) and anxiety measured by the Anxiety Subscale of Hospital Anxiety and Depression Scale (HADS) (*p* = 0.03). Interestingly, Bently et al. estimated the patient’s specific needs for occupational therapy interventions after lung cancer or MPM diagnosis [101]. The authors reported that more than half of patients suffering from thoracic cancer have occupational therapy needs assessed by the SPARC^©^ Questionnaire. However, to date, no studies have inquired about the effects of occupational therapy in patients with MPM. Intriguingly, Bayly et al. employed an early rehabilitative approach in lung cancer and MPM patients [102]. Feasibility and safety were reported as primary outcome measures, whereas promising effects in terms of HRQoL were underlined in the rehabilitation group. Lastly, Tanaka et al. carried out two studies [87,88] for evaluating the benefits of an early rehabilitation approach, providing cancer-specific data in MPM patients. The intervention protocol proposed in both studies consisted of an early mobilization five to six times a week starting the day after surgery (pleurectomy/decortication) and included sitting, standing, and walking. However, no home-based rehabilitation intervention was performed outside the hospital setting. In conclusion, despite some reports about the disease-specific effects of physical exercise in MPM patients [68,87], the small samples and the absence of comparison severely limit the strength of the results obtained by the studies previously reviewed.

### 4.4. Psychological Interventions

As widely evidenced in the whole cancer population, psychological, emotional, and social aspects of the disease should be adequately addressed in MPM patients also [89]. Indeed, the aggressive behavior and the poor prognosis characterizing MPM might be related to a significant burden in terms of emotional and psychosocial distress [103]. Precisely, about 19% of patients might experience anxiety, while 12.9% of patients might have symptoms of depression [104]. In this context, psychological symptoms should be treated with a combination of psychological and pharmacological approaches [104]. Moreover, given the widely evident adverse effects on pain and HRQoL of the disease, most of the multidisciplinary interventions proposed in the literature included healthcare professionals counseling or psychological support [68,90,91,93,100]. However, further studies on specific psychological interventions are needed to provide precise and tailored therapeutic strategies for MPM patients. On the other hand, a large consensus supports the efficacy of psychological interventions in the multidisciplinary management of MPM disabling symptoms in order to minimize the heavy emotional and social burden of this disease and optimize the effects of both conventional and complementary therapies. 

### 4.5. Pain Management

Pain is one of the most common symptoms complained about by patients suffering from MPM and could be the result of a complex combination characterized by nociceptive, neuropathic, and inflammatory factors [18]. Due to its multifactorial etiology, pain management in MPM patients is still challenging. The WHO’s Analgesic Ladder for Cancer Pain Relief is currently considered the cornerstone of cancer pain management [105]. Although this framework has been well defined, the doses and formulation of pain medications should be tailored to patients and cancer characteristics. A recent Cochrane systematic review reported that approximately 95% of cancer patients experiencing moderate to severe pain might have relief from mild to no pain within the first 14 days after opioid administration [106]. Moreover, a regular review of antalgic pharmacotherapy is strongly recommended to optimize pain relief and minimize side effects [107]. Despite this evidence, MPM patients might be poor responders to pharmacological therapies, especially when related to direct cancer invasion of the thoracic chest wall (i.e., costopleural syndrome) [107]. Against this background, adjuvant therapies should be considered, including tricyclic antidepressants or anti-epileptics. Unfortunately, the effects of specific drug combinations are still unclear, and patients should be carefully monitored to avoid side effects [108,109]. In patients not responding to pharmacological therapy, several non-pharmacological approaches have been proposed to manage pain in patients with MPM. First, radiotherapy is currently considered an effective intervention in palliative care, mainly in cases of bone erosion or cutaneous cancer involvement [107]. However, despite previous retrospective studies documenting the positive effects of radiotherapy in pain management in MPM patients [110,111], a recent RCT did not achieve a significant benefit of prophylactic radiotherapy following pleural interventions in MPM [112]. Moreover, a recent review underlined that palliative radiotherapy might be considered an effective approach in patients with good sensitivity, including non-sarcomatoid MPM subtypes, a performance status of 0 or 1, and a good EORTC performance index [113]. Although multiple therapeutic options were proposed as non-pharmacological approaches to treat MPM-associated pain, the spinothalamic tract ablation at C1/2 with radiofrequency is one of the most promising treatments [114]. This technique is known as cordotomy, and it seems related to significant benefits in terms of pain relief, medication administration, and sleep disturbances [115]. However, the scarce evidence collected up to now and the high skill level required severely limit its availability in most centers. In conclusion, the optimal and specific pain management framework in MPM patients still needs to be improved. A multidisciplinary approach should be considered in order to optimize pharmacological effects and potentially reduce adverse side effects of medications. This comprehensive treatment should include psychoeducational interventions, mind–body therapies, and physical exercise and should be considered in patients not fully responding to conventional therapies [116]. 

A summary of the above interventions can be found in Table 4. 

## 5. Future Perspectives

### 5.1. Tailored Multidisciplinary Rehabilitative Interventions

**Figure 2:**
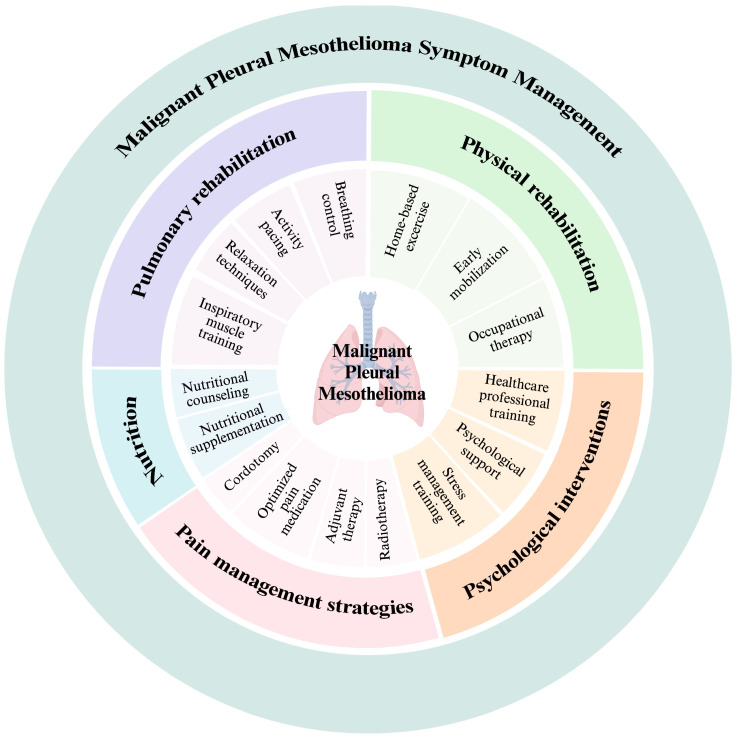
Malignant pleural mesothelioma comprehensive symptoms management.

Accumulating evidence has pointed out the crucial role of interdisciplinary and transdisciplinary rehabilitation interventions in addressing cancer-related symptoms [117]. Figure 2 summarizes the complementary treatments currently available to improve the symptom management of patients with MPM. 

On the other hand, it should be noted that most of the studies focusing on rehabilitative interventions in lung and MPM cancer patients described a comprehensive management, including respiratory interventions, activity pacing, pain management, and psychosocial support [90,91,93,100]. A multidisciplinary team management might have a role in the early detection of dyspnea or thoracic pain worsening, potentially implicated in the progression of the disease [118,119], with positive effects in follow-up improvement as reported for other tumors [120]. 

In addition, considering the recent advances in understanding the mechanisms underpinning chronic pain and the role of descending control projection, a tailored multidisciplinary rehabilitation framework might boost its efficacy in order to obtain optimal pain relief in MPM patients [121,122]. Specifically, a multitarget intervention might be effective at different levels of pain nervous circuits with possible positive implications in the modulation of pain chronicization pathways [121]. Conversely, multidisciplinary management of patients with MPM should start from a tumor-specific service model. Thus far, the interventions proposed were principally based on expertise exported from other chest tumors due to the low incidence of MPM and its poor prognosis [23]. As a result, MPM-specific rehabilitative indications are currently incomplete, and specific disabling symptoms are frequently underdiagnosed, underestimated, and undertreated. Thus, the evidence to guide clinicians in rehabilitative management of MPM patients was a bridging intervention with lung cancer treatment as a consequence of the gap in MPM-specific rehabilitative expertise. Indeed, only one study assessed the effectiveness of a novel model of dyspnea management in a heterogeneous sample including MPM patients [123]. In particular, the Breathlessness Intervention Service (BIS) is a multidisciplinary complex organization model that aims at improving breathiness in patients with advanced cancer by combining non-pharmacological and pharmacological approaches. The authors reported significant clinical advantages in addition to an improvement in cost-effectiveness compared to standard care. Despite these findings, there is still a gap of knowledge about the optimal multidisciplinary rehabilitative pathways of MPM patients. This issue is crucial to offer a specific standardized therapeutic pathway for MPM patients, reduce disability, and improve HRQoL. 

### 5.2. Possible Synergism between Physical Exercise, Immune System, and Immunotherapy

**Figure 3:**
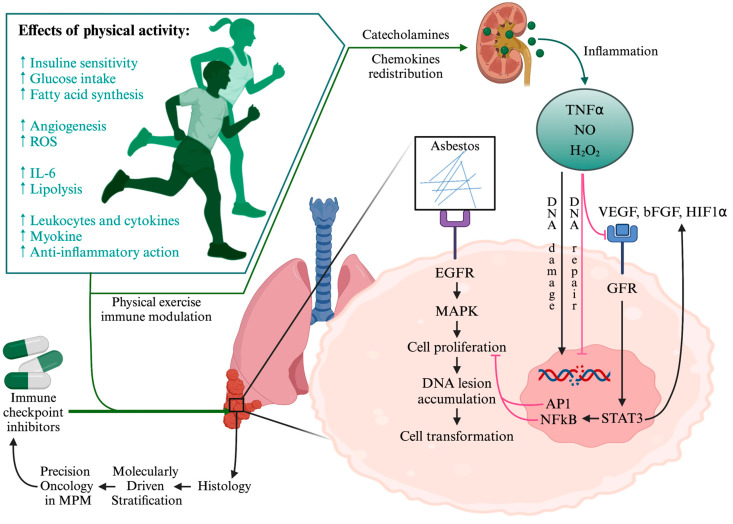
Physical activity-induced effects on malignant pleural mesothelioma oncogenesis, immune regulation, and immunotherapy.

The role of chronic inflammation in the oncogenesis process, promoting driver mutations or epigenetic mechanisms, has been widely documented [124,125,126]. In particular, the pathogenesis of MPM has been strictly connected to the chronic local inflammation triggered by asbestos fibers, inducing overexpression of VEGF, inactivation or mutations of several tumor suppressors (e.g., BAP1, CDKN2A, NF2, and TP53), chromosomal deletions, and epigenetic alterations [70]. Due to the pivotal involvement of inflammatory processes in MPM pathogenesis, their regulation has been suggested as a key factor in treating this cancer. Interestingly, immunotherapy is a novel and promising treatment to stimulate protective anti-tumor immunity [14,127,128,129]. Immune checkpoint inhibitors have been recently recommended as a well-tolerated therapeutic approach to improve outcome and survival in MPM patients. However, the results of the PROMISE-meso trial highlighted that the use of immune checkpoint inhibitors in unselected patients should be strongly reconsidered [130], underlining the need for specific predictive biomarkers. At the same time, the role of combined pharmacological therapies in MPM is far from being fully elucidated [131]. Lastly, given the crucial pathophysiological role of inflammation in MPM, dual blockage immune checkpoint inhibition has been proposed as well [132]. Besides the extensive research efforts in the immune–oncology field, recent years have seen an increasing interest in understanding immune response and tumor microenvironment modifications induced by physical exercise. A recent review reported that exercise might be associated with the regulation of immune response against tumors via acute mobilization of immune cells [133]. This hypothesis is supported by several preclinical studies suggesting that physical exercise in humans might have a positive role in regulating chemokine expression, promoting cytotoxic immune cell activity, and downregulating suppressor immune cells. These positive results could, to some extent, explain the reduction in cancer recurrence and cancer-specific mortality in patients with higher post-diagnosis levels of physical activity [134,135]. Nevertheless, several additional mechanisms have been suggested to explain the exercise-induced positive effects on cancer progression, including modulation of metabolic homeostasis and hormone levels, improvement in immune surveillance, and reduction in oxidative stress [136]. In this matter, the study by Kao et al. assessed the association between inflammatory markers and cancer-related symptoms and HRQoL in a homogeneous sample of MPM patients [71]. The authors highlighted a significant correlation between inflammation and HRQoL outcomes. Moreover, systemic inflammation was significantly related even with survival [71], suggesting a strict relationship between inflammation and MPM progression, thus highlighting the need for specific interventions to modulate the inflammatory response in these patients. Taken together, physical exercise and immune regulation might display a synergistic action not only in cancer prevention but also at the antitumoral treatment level [129,137]. In the era of precision medicine, providing specific data to enhance tailored treatments, including both pharmacological and non-pharmacological therapies, is becoming mandatory. The complex framework underpinning MPM oncogenesis, immunotherapy, and the immune regulation induced by physical activity have been summarized in Figure 3. 

These multimodal and synergistic interventions could boost the HRQoL outcomes in MPM patients. In the current scenario, also considering the recent COVID-19 pandemic, it is mandatory to have prognostic and predictive biomarkers in order to monitor specific biological modifications induced by different exercise modalities and to guide clinicians in prescribing tailored and targeted rehabilitative interventions [127,128,133,134,135,136,138,139,140]. 

## 6. Conclusions

This scoping review provides a comprehensive overview of the rehabilitation strategies and outcomes for patients with MPM. By synthesizing existing literature, we have identified key interventions such as pulmonary rehabilitation and early supportive care that significantly impact patients’ QoL and symptom management. In this context, our findings encourage the integration of these evidence-based practices into clinical guidelines to enhance the standard of care for MPM patients.

As with any scoping review, our study has limitations. While the majority of studies provided valuable insights into rehabilitation outcomes, there were variations in study designs, patient demographics, and outcome measures that may affect comparability. Some sources highlighted promising results with certain interventions, while others revealed ambiguous findings or even contradictory outcomes. These variations underscore the heterogeneity of MPM treatment approaches and the need for further research to establish consensus on optimal rehabilitation protocols. Moreover, the inherent scope of a scoping review limits our ability to conduct a formal quality assessment of included studies or to perform meta-analytical synthesis of data. Despite these limitations, our review provides a valuable synthesis of current knowledge, identifies gaps in the literature, and lays the groundwork for future research directions in MPM rehabilitation.

In conclusion, the present review showed that, despite substantial progress in understanding the mechanisms of MPM cancerogenesis, the overall management of MPM patients has not evolved significantly. Beyond conventional cancer approaches, there is a growing interest in rehabilitation and complementary therapies to improve functional outcomes and HRQoL. In this context, the present study advocates the need to implement patient-tailored rehabilitative intervention grounded in a comprehensive multidisciplinary strategy. This approach, being potentially synergistic with cancer treatments, might serve as a primary complementary intervention to enhance functional outcomes for individuals afflicted with MPM. As a final consideration, the lack of cancer-specific evidence in patients with MPM may influence our conclusions; thus, further good-quality studies are warranted in this scientific field to better investigate the rehabilitative management of this orphan oncological disease.

## Figures and Tables

**Table 1:** MPM prognostic and predictive factors.

Clinical Characteristics	Blood Test	Molecular Biomarkers
Age	Albumin	BAP1
Cancer-Directed Surgery	Hemoglobin	COX-2
Chemotherapy	LDH	EZH2
Chest Pain	NLR	FGF-binders
Gender	PLT	MET
Histologic Diagnosis	WBC	PD-L1
Histologic Type		SMRP
Pleural Involvement		TSP-1
PS		VEGF
Race		
Stage		
Weight Loss		

Abbreviations: COX-2: cyclooxygenase-2; FGF: fibroblast growth factor; LDH: lactate dehydrogenase; NLR: neutrophil-to-lymphocyte ratio; PD-L1: programmed death-ligand 1; PLT: platelet; PS: performance status; SMRP: serum-soluble mesothelin-related peptide; TSP-1: thrombospondin-1; VEGF: vascular endothelial growth factor; WBC: white blood cell.

**Table 2:** Malignant pleural mesothelioma therapeutic interventions.

Systemic Therapy	Radiotherapy	Surgery
Cisplatin and pemetrexed	Adjuvant	Talc pleurodesis
Carboplatin and pemetrexed	Palliative	EPP
Nivolumab and ipilimumab		PD
Additional bevacizumab		EPD
TTF		
Ramucirumab and gemcitabine		
Nivolumab		

Abbreviations: EPD: extended pleurectomy decortication; EPP: extra-pleural pneumonectomy; PD: pleurectomy decortication; TTF: tumor-treating fields.

**Table 3:** Main disabling sequelae.

Fatigue	Early and Severe Symptom Burden
	Significantly reduces HRQoL
Cough	Impacts physical and social functioning
Dyspnea	Associated with worse overall HRQoL
Chest Pain	Negatively affects physical and emotional functioning
Lethargy	Leads to reduced activity levels
	Contributes to poor HRQoL
Weight Loss	Impairs physical health and HRQoL

Abbreviations: HRQoL: health-related quality of life.

**Table 4:** Quality of life and rehabilitative interventions.

Quality of Life, Functioning, and Disability in MPM Patients
38% malnourished and 54% pre-sarcopenic
Malnutrition decreases HRQoL
Malnutrition increases mortality risk
Fatigue correlated with inflammatory markers; affects HRQoL and overall survival
Induction chemotherapy improves FEV1 and VO2 peak
Surgery (EPP) with adjuvant therapies shows mixed results on functional tests
Physical function metrics correlate with mortality
Respiratory interventions
Comprehensive intervention improves breathlessness, PS, physical/emotional states
Significant improvements in breathlessness, functional capacity, activity levels, distress, fatigue, emotional function, and depression
Three-session group showed potential benefits for breathlessness severity and distress
One session per week may be cost-effective in symptom management
Physical exercise interventions
Exercise and stress management improve anxiety and depression in chemotherapy patients
Home-based exercise improves 6MWT in the MPM subgroup
Early rehabilitative approach feasible and safe, potential HRQoL benefits
Early mobilization post-surgery shows potential benefits but lacks home-based intervention data
Psychological interventions
Significant burden of anxiety (19%) and depression (12.9%) in MPM patients
Supportive counseling/psychological support included in interventions
Pain management
WHO Analgesic Ladder is an effective framework; 95% of cancer patients find pain relief with opioids within 14 days
Radiotherapy is effective in pain management, especially with bone erosion/cutaneous involvement
Cordotomy is promising for pain relief, medication reduction, and sleep disturbances
Multidisciplinary approach needed, including psychoeducational and mind–body therapies

Abbreviations: EPP: extra-pleural pneumonectomy; FEV1: forced expiratory volume in the first second; HRQoL: health-related quality of life; MPM: malignant pleural mesothelioma; PS: performance status; WHO: World Health Organization; 6MWT: 6-minute walking test.

## Data Availability

The data presented in this study are available upon request from the corresponding author.
